# Endothelial progenitor cells contribute to the development of ovarian carcinoma tumor blood vessels

**DOI:** 10.3892/ol.2014.1917

**Published:** 2014-02-26

**Authors:** DORIN GRIGORAS, LAURENŢIU PIRTEA, RALUCA AMALIA CEAUSU

**Affiliations:** 1 Department of Obstetrics and Gynecology, ‘Victor Babeş’ University of Medicine and Pharmacy, Timişoara 300041, Romania; 2 Department of Microscopic Morphology, Angiogenesis Research Center, ‘Victor Babeş’ University of Medicine and Pharmacy, Timişoara 300041, Romania

**Keywords:** ovarian carcinoma, endothelial progenitors cells, angiogenesis

## Abstract

Only a few studies in the literature have reported the contribution of endothelial progenitor cells (EPCs) in ovarian tumors, and with regard to malignant tumors, the data on the pre-existing endothelium insertion rate and the extent to which these cells contribute to tumor angiogenesis is controversial. The present study demonstrated the existence of EPCs and evaluated the expression of two markers, AC133 (also known as cluster of differentiation 133 or prominin) and tyrosine kinase with immunoglobulin-like and EGF-like domains 2 (Tie2), signaling the presence of EPCs in the pre-existing endothelium. In total, 62 female patients who were diagnosed with ovarian tumors were retrospectively selected over a four-year period. Immunohistochemical analyses used Tie2 and AC133 as primary antibodies. In total, 27.4% of ovarian tumor cases expressed AC133 and Tie2 in blood vessel endothelial cells. The expression of these two markers did not correlate with the clinicopathological prognostic parameters, histological type, vascular microdensity or vessel type. The expression of AC133 and Tie2 in blood vessel endothelial cells contributes to angiogenesis progression in cases where the budding process is reduced or absent, as shown by the inverse correlation with the rate of proliferation of the endothelial cells.

## Introduction

Endothelial progenitor cells (EPCs) are bone marrow-derived cells that can be found in peripheral and umbilical cord blood. The cells were first isolated in the study by Asahara *et al* (1997), where it was demonstrated that cluster of differentiation 34-positive (CD34^+^) hematopoietic progenitor cells from adults can differentiate *ex vivo* into the endothelial phenotype (1). These cells express endothelial markers and are incorporated into the neoformation vessels in ischemic areas. Data in the literature have supported the presence of circulating hemangioblasts in adults, and EPCs are defined as CD34- and VEGFR2-expressing elements (2,3). CD133, also known as prominin or AC133, is a conserved antigen with unknown biological activity, which is expressed by hematopoietic stem cells, but is absent in mature endothelial cells and in the monocyte line (4). Under these conditions, CD133^+^/VEGFR2^+^ cells are likely to reflect immature progenitors and the cells interspersed in the vascular endothelium.

In the group of circulating blood mononuclear cells there may be several sources of EPCs, including hematopoietic stem cells, myeloid cells that can differentiate on endothelial cells by growing, other progenitor circulating cells and mature endothelial circulating cells. The first evidence of the existence of several circulating EPCs was reported by Lin *et al* (5).

Although the existence of EPCs has been demonstrated, with regard to malignant tumors the data is controversial on the pre-existing endothelium insertion rate and the extent to which these cells contribute to tumor angiogenesis. From these points of view, the results obtained so far vary between the extremely wide limits of 0 and 72 % for various human tumors. So far, no such studies have reported the contribution of EPCs in ovarian tumors. For this reason, the present study evaluated the expression of two markers, AC133 and tyrosine kinase with immunoglobulin-like and EGF-like domains 2 (Tie2), which signal the presence of EPCs in the pre-existing endothelium.

## Materials and methods

### Patient selection

In total, 62 female patients who were diagnosed with ovarian tumors were retrospectively selected over a four-year period. The patients had complete clinicopathological and post-surgical evaluation data, and were well characterized with regard to the invasion (local and distant) and surgical protocols. Signed consent was obtained from each patient. All procedures were carried out according to the principles embodied in the Declaration of Helsinki and were approved by the Institutional Review Board of ‘Victor Babeş’ University of Medicine and Pharmacy, Timişoara, Romania.

### Specimens and histopathological primary processing

Tumor specimens were surgically removed and the most representative sections were carefully selected to include tumor and adjacent normal ovarian tissues. Tumor sections with necrosis and extensive hemorrhages were avoided. Small tumor tissues (10×10×3-mm biopsies) were washed in saline solution, fixed in 10% buffered formalin for 24 h and then paraffin embedded. For each paraffin-embedded specimen, 5-μm serial sections were mounted on silanized slides. One slide from each case was stained with hematoxylin and eosin using a routine method for histopathological evaluation and also for case selection for the immunohistochemical procedures.

### Immunohistochemistry

Heat-induced epitope retrieval was performed with a citrate-based solution (pH 6.0; Novocastra Laboratories, Ltd., Newcastle upon Tyne, UK) for 30 min. Endogenous peroxidase blocking was carried out with 3% hydrogen peroxide for 5 min, followed by incubation for 30 min with Tie2 (dilution 1:300, mouse monoclonal clone 9; Santa Cruz Biotechnology, Inc., Santa Cruz, CA, USA) and AC133 (dilution 1:300, rabbit polyclonal clone H-284; Santa Cruz Biotechnology, Inc.) as primary antibodies. The Bond Polymer Refine Detection System (Leica Biosystems, Newcastle upon Tyne, UK) was used for visualization. 3,3 Diaminobenzidine dihydrochloride was applied as a chromogen and hemotoxylin was used as a counterstain. The entire immunohistochemical procedure was performed with the Leica Bond-Max autostainer (Leica Biosystems).

## Results

Upon microscopic evaluation of the hematoxylin and eosin-stained tumor specimens, four main histopathological types of ovarian tumors were identified: Serous carcinomas (62%), mucinous carcinomas (18%), clear cell carcinomas (6%) and ovarian germ cells tumors (8%) and undifferentiated carcinomas (6%). The majority of the aforementioned ovarian tumors exhibited a G2 tumor grade (58%), followed by grades G3 (39%) and G1 (3%).

In evaluating AC133 and Tie2 expression, the location of the positive cells was examined and only elements with a positive cytoplasmic reaction that defined the lumens of the blood vessels were subjectively assessed. AC133 was positive in 18 out of 62 specimens (29.03%), and Tie2 was positive in 21 of the specimens (33.87%). Co-expression of the markers was noted in 17 cases (27.42%), in which it was considered that the positive reaction reflected the insertion of the endothelial progenitor cells into the pre-existing endothelium. The presence of endothelial progenitor cells did not exhibit a statistically significant correlation with vascular microdensity, vessel type or histopathological form.

**Figure 1:**
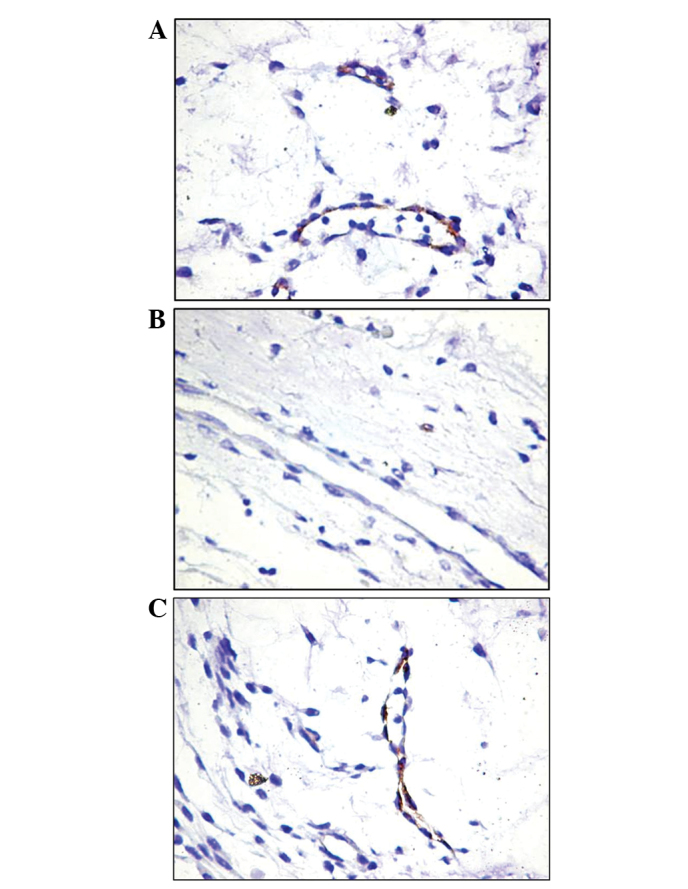
Intratumoral area with small vessels positive for AC133. (A) Peritumoral area with a mature negative vessel and (B) a small positive vessel. (C) The heterogeneous distribution of the positive reaction for AC133 (magnification, ×400).

For the expression of AC133, the positive reaction was constantly evident in the vessels of the tumor area. These vessels were small, and relatively frequent positive endothelial cells lined the majority of the lumens (Fig. 1A). Notably, the endothelial cells were the only AC133-positive cells in the majority of the tumor stroma cases. In the peritumoral area, the blood vessels were predominantly AC133-negative, particularly when their morphology was indicative of a mature character. Occasionally, in extremely small vessels, a positive reaction was observed (Fig. 1B). The most frequently observed aspect in the intratumoral area was the heterogeneous model with alternating AC133-positive and -negative cells (Fig. 1C).

**Figure 2:**
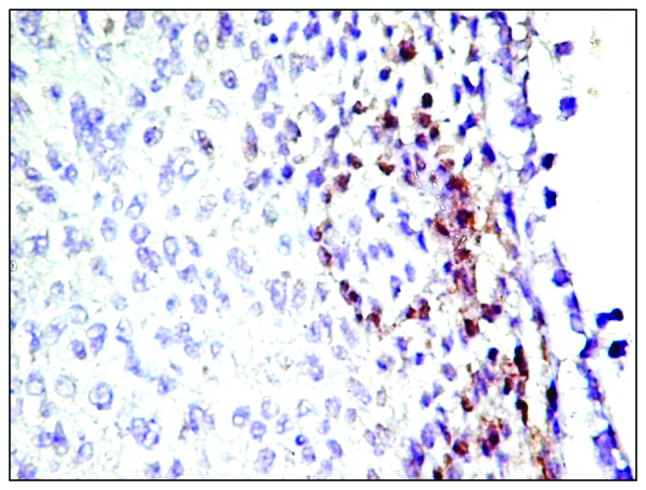
Tumor cells positive for AC133, located at the level of the proliferation line (magnification, ×400).

In only two out of the 62 cases, AC133-positive neoplastic cells were focally observed in the intratumoral area. The distribution pattern of the positive reaction was diffuse, cytoplasmic and not predominantly in the membrane (Fig. 2). These cells formed a distinct population of tumor cells, preferentially located at the tumor proliferation front, which could represent tumor stem cells. In the present study tumor stem cells were positive for this marker, but the method of detection is not specific enough and further studies are required to demonstrate their character.

**Figure 3:**
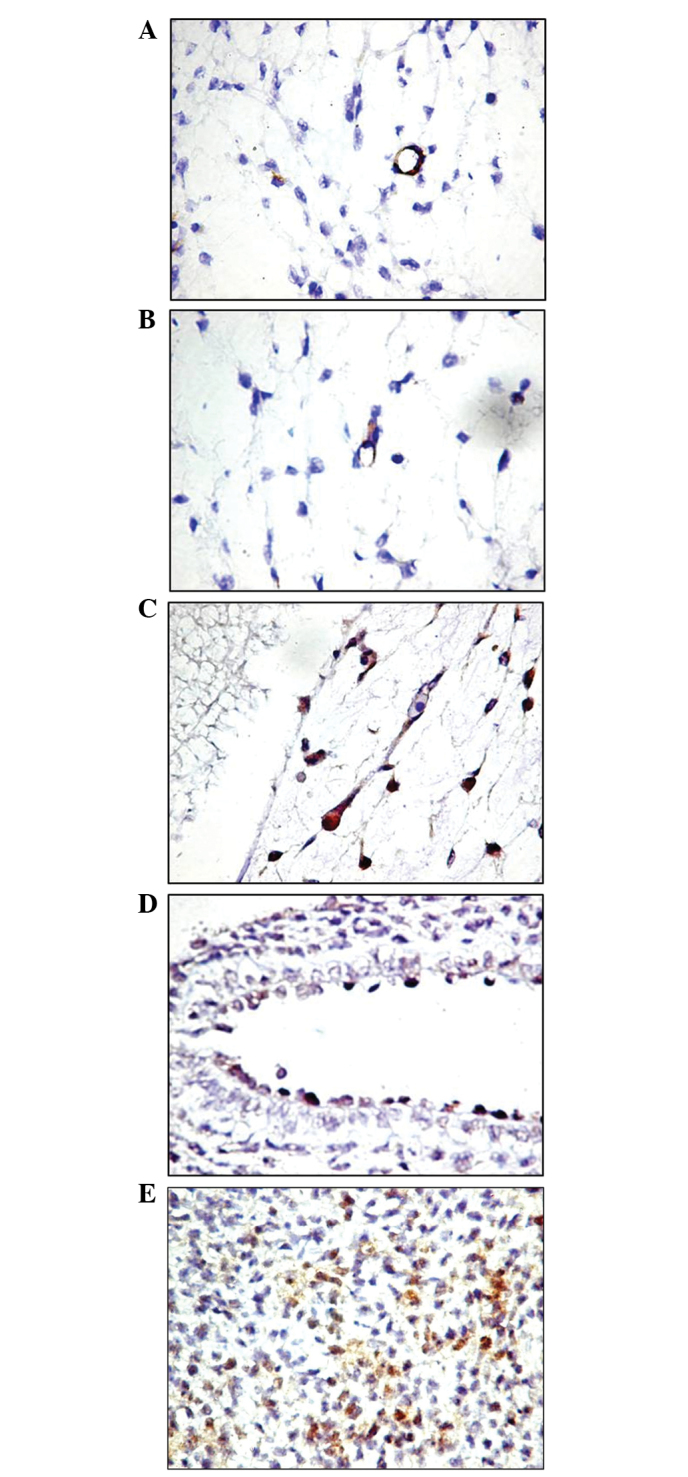
Immunoreaction for Tie2 in ovarian tumor cells. (A, B) Homogeneous distribution pattern in the small vessels of the tumor area. (C) Heterogeneous distribution pattern. (D) Positive reaction in a pre-existing vessel. (E) Positive reaction in the tumor cells (magnification, ×400).

The immunoreaction for Tie2 was also selective for cells that defined the blood vessel lumens. Even under these conditions, a small number of vessels with Tie2-positive endothelial cells were identified in the tumor area, and the distribution model was found to be homogeneous in the small vessels (Fig. 3) and heterogeneous in the larger vessels with relatively large lumens (Fig. 3C). Unlike the reaction for AC133, Tie2 expression was positive in the endothelium of pre-existing mature blood vessels, which were larger in size (Fig. 3D). The immunoreaction was found to be restricted to the endothelium and did not stain perivascular cells. Since it was not possible to quantify the Tie2-positive cells compared with the Tie2-negative cells at the endothelial level, based on subjective observations it appears that Tie2 is less selective in identifying EPCs, and this most likely indicates the presence of pre-existing activated endothelial cells. The two cases in which AC133-positive tumor cells were identified were also Tie2-positive, but the number of positive cells was significantly higher.

## Discussion

Tumor neovascularization represents a key point in tumor progression, and has been extensively demonstrated to result from the process of angiogenesis (6). The role ascribed to the cancer cells during the process of tumor angiogenesis is the initiation of the angiogenic switch, which is a critical step in tumor progression (7).

Treatment for ovarian cancer is now shifting from conventional chemotherapy to molecular-targeted therapies (8). An example of one such therapy is the inhibition of the specific cytokines essential for tumor vascularization (9). Antiangiogenesis therapy has thus become a novel strategy for ovarian cancer treatment.

Su *et al* (2010) demonstrated that the levels of EPCs are significantly increased in the blood of patients with ovarian cancer and are correlated with cancer stage and residual tumor size (8). It was also shown that treatment reduces the levels of circulating EPCs in patients. Previous clinical correlations have shown that a positive correlation occurs between an increase in EPC circulation in pancreatic, breast and ovarian cancer patients, and tumor stage and size (10,11). The co-expression of AC133 and Tie2 occurred in 27.4% of cases in the present study. Bagley *et al* (2011) revealed that tumor endothelial marker 7 (TEM-7) is a vascular protein associated with angiogenic status and that it may be a novel and attractive target for antiangiogenic therapy (12).

The tumor microenvironment plays a significant role in the activation of circulating EPCs and the mediation of neovascularization. Stressors, including hypoxia, glucose deprivation and reactive oxygen species, are activated in the tumor microenvironment and result in the upregulation of the transcription of angiogenic factors, including vascular endothelial growth factor (VEGF), stromal cell-derived factor 1 monocyte chemotactic protein-1 and erythropoietin, in EPCs (13–15). In the present study it was noticed that in the majority of tumor stroma cases, the endothelial cells were the only cells positive for AC133.

EPCs are regarded as bone marrow-derived cells that are able to migrate into the peripheral blood in response to cytokines, such as VEGF (16). As opposed to in ischemic conditions, the role of circulating EPCs in tumor growth and angiogenesis is not clear. EPCs have been identified as a potential marker for the response to antiangiogenic therapies and neovascularization, and they also possess a high proliferation potential (17).

Initially, Tie2 was found to be overexpressed in tumoral vessels, and it is also expressed in several types of cancer, including leukemia, and solid neoplasms, including gliomas and gastric and breast tumors. Tie2 expression in various tumoral compartments highlights this cellular receptor as an attractive target for cancer therapy (18).

In summary, the results of the present study revealed that 27.4% of ovarian tumor cases express AC133 and Tie2 in blood vessel endothelial cells. The expression of these two markers did not correlate with any clinicopathological prognostic parameters, including histological type, vascular microdensity and vessel type. Co-expression of the markers most likely reflects the insertion of endothelial progenitor cells into the pre-existing endothelium. This phenomenon contributes to angiogenesis progression in cases where the budding process is reduced or absent, as shown by the inverse correlation with the rate of endothelial cell proliferation.
